# Obstacles and Coping Strategies of CAR-T Cell Immunotherapy in Solid Tumors

**DOI:** 10.3389/fimmu.2021.687822

**Published:** 2021-05-19

**Authors:** Lele Miao, Zhengchao Zhang, Zhijian Ren, Futian Tang, Yumin Li

**Affiliations:** ^1^ Department of General Surgery, Second Hospital of Lanzhou University, Lanzhou, China; ^2^ Key Laboratory of the Digestive System Tumors of Gansu Province, Second Hospital of Lanzhou University, Lanzhou, China

**Keywords:** chimeric antigen receptor T cell, immunotherapy, obstacles, coping strategies, solid tumors

## Abstract

Chimeric antigen receptor (CAR) T-cell immunotherapy refers to an adoptive immunotherapy that has rapidly developed in recent years. It is a novel type of treatment that enables T cells to express specific CARs on their surface, then returns these T cells to tumor patients to kill the corresponding tumor cells. Significant strides in CAR-T cell immunotherapy against hematologic malignancies have elicited research interest among scholars in the treatment of solid tumors. Nonetheless, in contrast with the efficacy of CAR-T cell immunotherapy in the treatment of hematologic malignancies, its general efficacy against solid tumors is insignificant. This has been attributed to the complex biological characteristics of solid tumors. CAR-T cells play a better role in solid tumors, for instance by addressing obstacles including the lack of specific targets, inhibition of tumor microenvironment (TME), homing barriers of CAR-T cells, differentiation and depletion of CAR-T cells, inhibition of immune checkpoints, trogocytosis of CAR-T cells, tumor antigen heterogeneity, etc. This paper reviews the obstacles influencing the efficacy of CAR-T cell immunotherapy in solid tumors, their mechanism, and coping strategies, as well as economic restriction of CAR-T cell immunotherapy and its solutions. It aims to provide some references for researchers to better overcome the obstacles that affect the efficacy of CAR-T cells in solid tumors.

**Graphical Abstract:**
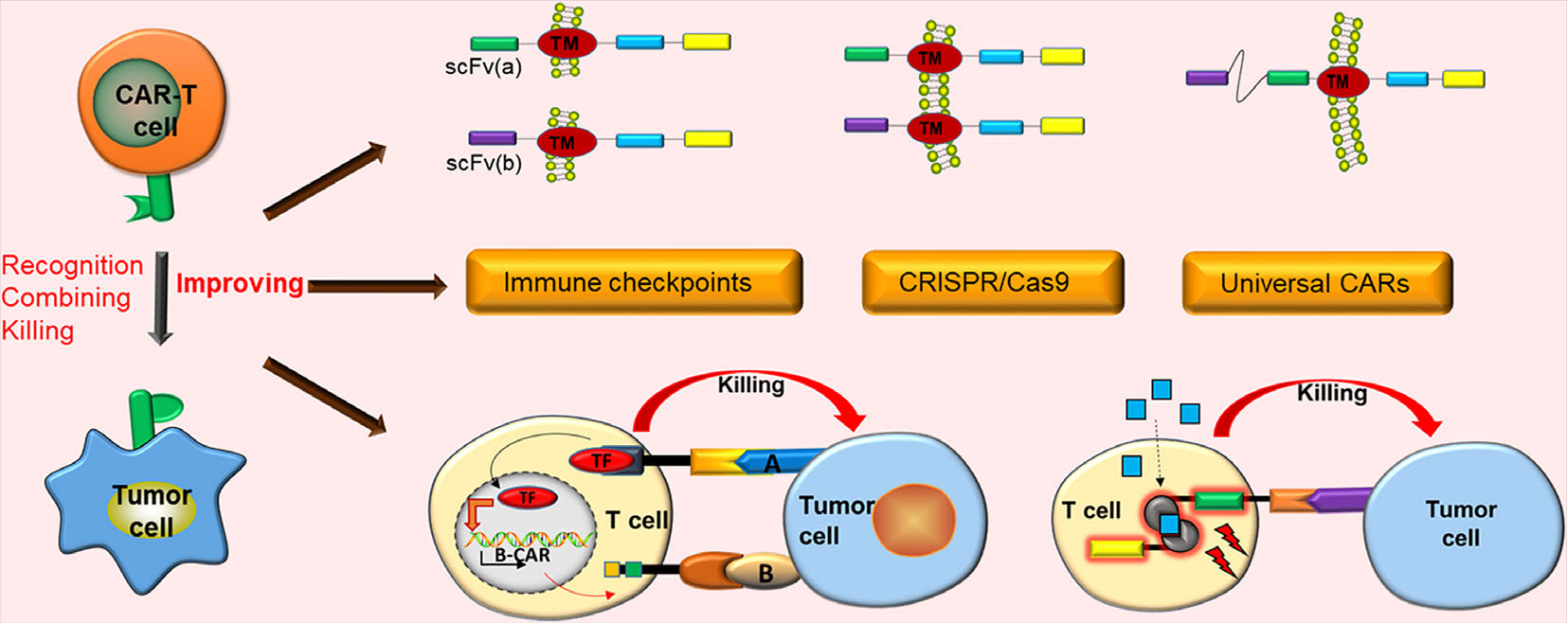
CAR-T cell immunotherapy has demonstrated notable future opportunities in the mainstream cancer treatment of solid tumors. However, it still has numerous obstacles in the treatment of solid tumors. Only by adopting effective strategies against these obstacles can CAR-T cells exert better curative effects in the treatment of solid tumors.

## Introduction

Malignant tumors present a serious global health issue with increasing annual incidence and high mortality rates. At present, the available treatment approaches for malignant tumors include surgery, chemotherapy, radiotherapy, targeted therapy, and immunotherapy. Among them, immunotherapy has gradually evolved in recent years. In 2012, 6-year-old Emily Whitehead diagnosed with acute lymphoblastic leukemia (ALL) received CD19-CAR-T cell therapy. Thereafter, the patient achieved complete remission (1). Emily’s story elicited an upsurge in tumor immunotherapy and simultaneously opened the prelude to CAR-T cell immunotherapy for tumor treatment.

### The Structure and Principle of CAR-T Cells

**Figure 1:**
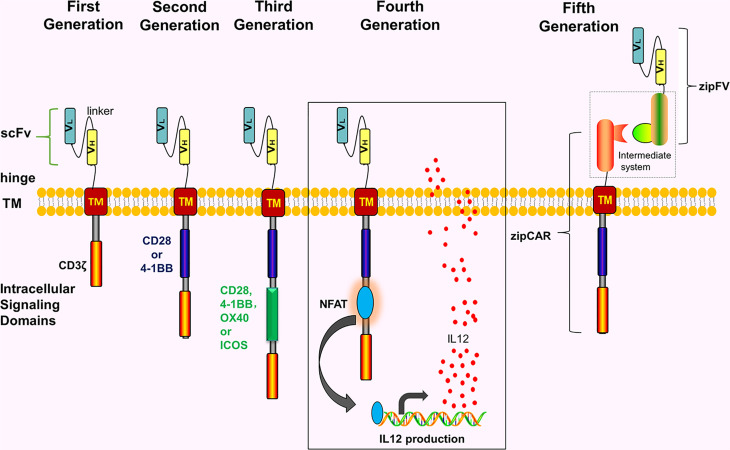
The development of the CARs. The intracellular structure of first-generation CARs only has one signal structure domain (CD3ζ) without co-stimulatory molecules. The second-generation CARs add one co-stimulatory molecule to the first-generation CAR, e.g, CD28, 4-1BB, OX40 or ICOS. The third-generation CARs contain 2 costimulatory molecules. The fourth-generation CARs are modified by adding NFAT or suicide genes based on the second-generation CARs or the third-generation CARs. The fifth-generation CARs use a “third-party” intermediate system to separate the antigen-binding domain of CARs from the T cell signaling unit.

CAR comprises 4 parts including single-chain variable fragment (scFv), extracellular hinge region, transmembrane region, and intracellular signaling domain (immunoreceptor tyrosine-based activation motif, ITAM) (**Figure 1**). Notably, extracellular hinge region and scFv are also referred to as extracellular target binding domain. The extracellular target binding domain recognizing tumor antigens and intracellular signaling domain are recombined *in vitro* to form recombinant plasmids, then transfected into T cells of the patient with transfection technology *in vitro*, hence these T cells express CARs. Subsequently, these T cells are expanded and screened *in vitro*, eventually returning to the body. Since its initial proposal in 1989, CAR has so far developed to the 5^th^ generation (**Figure 1**).

### The Clinical Application of CAR-T Cell Immunotherapy

CAR-T cell immunotherapy has demonstrated significant curative effects in the treatment of hematologic malignancies. These significant achievements promote its application in the treatment of solid tumors. In contrast with the therapeutic effect of CAR-T cell immunotherapy in hematological malignancies, the general curative effect on solid tumors is insignificant. Therefore, CAR-T cell immunotherapy has great potential in the treatment of solid tumors, providing a novel idea and method for the clinical treatment.

## Obstacles and Coping Strategies of CAR-T Cell Immunotherapy in the Treatment of Solid Tumors

Significant achievement of CAR-T cell immunotherapy in hematologic malignancies has promoted its application in the treatment of solid tumors. Therefore, several trials currently apply CAR-T against solid tumors, including glioblastoma (2), lung cancer (3), liver cancer (4), gastric cancer (5), renal cancer (6), prostate cancer (7), etc. Nevertheless, based on a meta-analysis on the efficacy of CAR-T in treating solid tumors, CAR-T cell immunotherapy produced a comprehensive response rate of 9% (8). This is primarily attributed to various obstacle factors, as discussed below.

### Lack of Tumor-Specific Antigens (TSAs)

Lack of TSAs in solid tumors is one of the primary reasons for the poor efficacy of CAR-T cell immunotherapy. In most of the current solid tumors, treatment targets are mostly tumor-associated antigens (TAAs), thus the specificity is not high. This triggers inevitable off-target effects of CAR-T cells in the treatment of solid tumors. Off-target effects sometimes cause severe adverse reactions and even life-threatening outcomes. In one clinical trial, researchers constructed carboxy-anhydrase-IX (CAIX)-targeted CAR-T cells to treat patients diagnosed with advanced renal cell carcinoma (RCC) (9). As a consequence, 4 out of 12 patients were terminated in the experiment because of severe liver damage. This was attributed to CAIX-CAR-T cells damaging the bile duct epithelial cells that express CAIX.

Notably, designing CARs for TSAs is an effective approach to solve the off-target effect of CAR-T cell immunotherapy. Nonetheless, these TSAs are extremely rare, therefore, TAAs have to be utilized. Therefore, it is essential to institute measures that enhance the efficiency of CAR-T cells binding to solid tumor cell surface antigens.

#### Combined CAR-T Cell Immunotherapy

**Figure 2:**
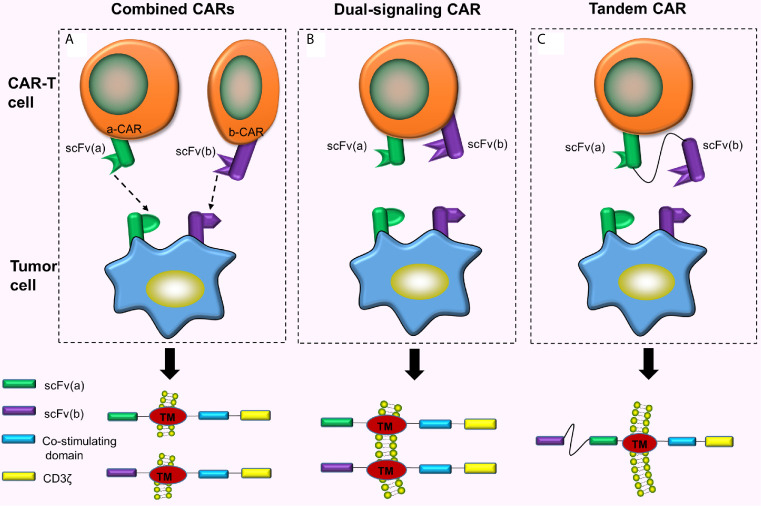
Combined CARs, Dual-singling CAR and Tandem CAR. **(A)** Simultaneous or sequential use of 2 or more CAR-T cells treating a similar malignant tumor; **(B)** Dual-signaling CAR refers to the simultaneous and separate expression of 2 different CARs on similar T cell surface; **(C)** Two different scFvs are connected hand in hand to form tandem CAR on the surface of the T cell.

Combined CAR-T cell immunotherapy refers to the simultaneous or sequential use of 2 or more CAR-T cells for a similar malignant tumor (**Figure 2A**). This can exhaustively expend TAAs and enhance the ability of CAR-T cells to recognize and bind to the corresponding tumor cells. Feng et al. (10) treated one patient with advanced unresectable/metastatic cholangiocarcinoma (CCA) with a CAR-T cocktail immunotherapy. This therapy involves continuous infusion of epidermal growth factor receptor (EGFR)-CAR-T cells and CD133-CAR-T cells, respectively. As a result, the patient achieved a 13-month partial response (PR). This strategy suggested the feasibility of CAR-T cocktail immunotherapy in the treatment of solid malignancies. The combined use of 2 different CAR-T cells has better efficacy compared to using either CAR-T cell alone (11). However, the pros and cons of this therapy in the prognosis of cancer patients and toxic reactions produced during the treatment still require numerous clinical trials for verification and evaluation. Additionally, reports suggest another combination mode, called dual signaling CAR, i.e., 2 different CARs simultaneously and separately expressed on a similar T cell surface (12, 13) (**Figure 2B**). One study has revealed that dual-signaling CAR-T cells have better antitumor activity compared to one type of CAR-T cells or 2 types of hybrid CAR-T cells *in vivo* (12). Also, since the 2 CARs need to be separately constructed, both time and cost remain relatively increased.

#### Tandem CARs (TanCARs)

TanCARs adopt the design philosophy of the “OR” gate. Down-regulation or mutation of target antigens is often observed in cancer cells, and the loss of target antigens causes cancer cells to escape eventually inducing tumor recurrence (14). Therefore, if TAAs on the tumor cell surface are fully utilized and 2 or more TAAs simultaneously targeted, tumor recurrence caused by target antigen loss can be largely prevented (because the probability of losing both antigens is much lower than the probability of losing one antigen). Based on this principle, Grada et al. (15), designed a bispecific CAR, tanCAR, which connected 2 different scFvs in tandem to a single transgenic receptor (**Figure 2C**). This series connection allows TanCAR subunits to freely move (15). The tandem CAR-T cell contains 2 different scFvs. CAR is activated when any scFv binds to the tumor cell surface antigen. When both scFvs simultaneously bind to the 2 antigens on the tumor cell surface, the CAR is activated with a synergistic effect which further enhances the activation and tumor-killing ability of CAR-T cells (15, 16).

Generally, unlike single antigen-specific CAR or a combination of 2 single antigen-specific CARs, tanCAR effectively prevents the escape of tumor cells and enhances tumor-killing effects with less toxicity (15, 16). Moreover, researchers further optimized the CAR by developing a specific type of trivalent CAR-T cells, which simultaneously express 3 CAR molecules, targeting HER2, IL13Rα2, and EphA2 on the surface of glioblastoma cells. The trivalent CAR-T cells revealed greater anti-glioma activity in mouse models (17). In recent years, tanCARs have been increasingly applied in the research and treatment of solid tumors.

#### The synNotch AND Gate T Cells (AND Gate CARs)

**Figure 3:**
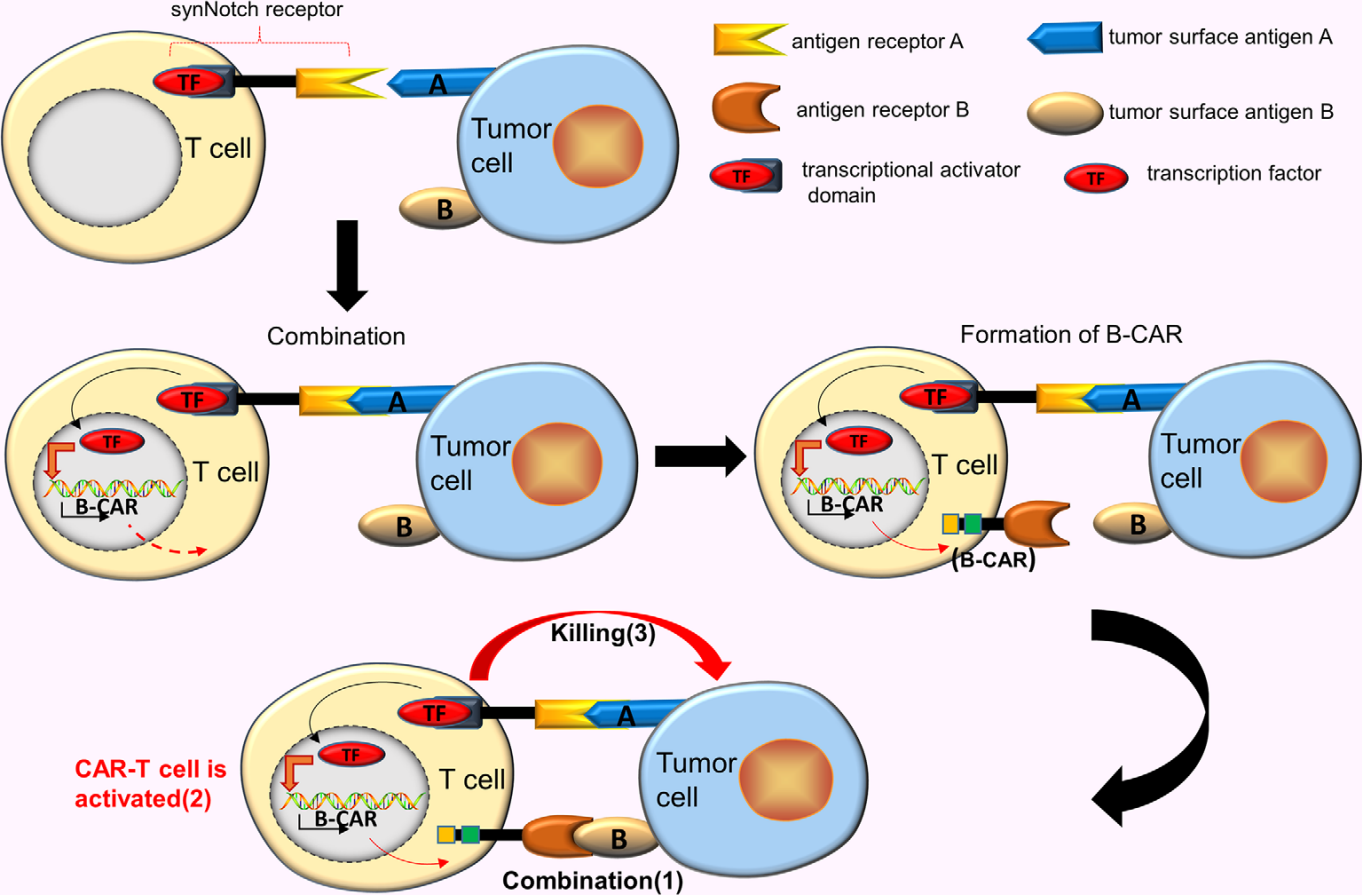
AND gate CAR. SynNotch receptor uses the scFv to bind to the corresponding target antigen A, however, the combination of 2 does not trigger the T cells activation. This binding causes the synNotch receptor to split and release the transcriptional activator domain, which enters the nucleus and drives the expression of another B-CAR gene. Subsequently, the surface of the T cell will express B-CARs. At this time, the newly expressed CARs binds to the target antigen B, activating the T cells.

CAR-T cells designed for TAAs inevitably produce off-target effects. As such, improving the activity and accuracy of CAR-T cells is an ideal improvement method. Roybal et al. (18) developed a new class of modular receptors, called synNotch receptors. These receptors use the scFv in binding to the corresponding target antigen A, however, the combination of two does not trigger the T cells activation, unlike the conventional CARs. Instead, this binding causes the synNotch receptor to split and release the transcriptional activator domain, which enters the nucleus and drives the expression of other B-CAR genes. Subsequently, the surface of the T cell express B-CARs against the target antigen B. At this time, the newly expressed CARs binds to the target antigen B, eventually activating the T cells (18) (**Figure 3**).

A few studies reveal that the synNotch AND gate T cells have shown precise recognition and strong tumor-killing ability, which are ineffective for tumor cells expressing a single antigen (can reduce off-target effects), and effectively eliminating tumor cells expressing the corresponding double antigens (19). Research proposes one assumption that combines synNotch receptor with tanCAR in constructing a new type of CAR-T cells that simultaneously eliminate tumor cells expressing 3 corresponding antigens (20), a hypothesis has recently been tested. Williams et al. (21) effectively constructed this new type of CAR-T cell by transcriptionally linking multiple receptors. These CAR-T cells comprised synNotch receptors targeting hepatocyte growth factor receptor (MET), and tanCARs targeting EGFR and HER2. This new design improved the ability of CAR-T cells to recognize and kill targeted tumor cells (simultaneously expressing [HER2 and/or EGFR] and MET).

#### Universal CARs

Notably, the fifth-generation CARs are also called universal CARs. These CARs use a “third party” intermediate system to separate the antigen-binding domain of CAR from the T-cell signaling unit, which theoretically recognizes different antigens without reinventing the T-cell to recognize new antigens (22–25) (**Figure 1**). In theory, universal CARs minimize tumor cell escape. Universal CARs comprise BBIR (biotin-binding immune receptors) CAR (23) and SUPRA (split universal and programmable) CAR (25). The BBIR CAR system (23) comprises 2 parts, i.e., the BBIR containing dimeric avidin, which binds to the membrane of T cells; and biotin, which binds to antigen-specific molecules (e.g, scFv, Abs, or tumor-specific ligands). The 2 are combined by non-covalent action to establish the targeted relationship between T cells and tumor antigens and finally activating T cells. Also, the SUPRA CAR system (25) comprises 2 parts, i.e., T cell universal receptor with leucine zipper adaptor (zipCAR) and scFv with leucine zipper adaptor (zipFV) targeting tumor-specific antigens. The targeted relationship between T cells and tumor antigens is established through a combination of zipCAR and zipFV. And T cells finally are activated. In theory, universal CARs should be the ideal CARs.

### Tumor Microenvironment (TME) Inhibits CAR-T Cell Activity

Tumor microenvironment (TME) refers to the survival environment of tumors. Its components primarily include cancer cells, surrounding immune cells, cancer-associated fibroblasts, stromal tissues, microvasculars, hormones, cytokines and chemokines, etc. (26). TME disrupts and inhibits the activity of CAR-T cells in the treatment of solid tumors, causing the inability of CAR-T cells to effectively recognize and kill tumor cells (27). Therefore, this situation can be enhanced in two ways,i.e., (a) First, based on the characteristics of TME, CAR-T cells that maintain activity and resist immunosuppressive factors in TME can be designed and enhanced; (b) Secondly, remodeling TME to minimize its immunosuppressive effect.

#### Designing CAR-T Cells Based on the Characteristics of TME

The glycolytic metabolism of cancer cells causes hypoxia, low pH, and low nutrition in TME, which inhibit the activity of CAR-T cells (28, 29). For instance, Juillerat et al. designed hypoxia-inducible factors 1-alpha (HIF1α)-CAR-T cells based on the characteristic of hypoxia in TME. In contrast with normal oxygen concentration, the ability of these CAR-T cells to kill their target cells in a hypoxic environment was significantly enhanced (30). Therefore, CARs can also be designed based on the low pH and nutritional characteristics of TME.

#### Improving CAR-T Cells Based on Immunosuppressive Cells or Molecules

The solid tumor microenvironment is rich in suppressive immune cells, including regulatory T cells (Tregs), myeloid-derived suppressor cells (MDSCs), tumor-associated neutrophils (TANs), etc. These suppressive immune cells secrete growth factorβ (TGF-β), IL-10, indoleamine-2, 3-dioxygenase (IDO), etc. which inhibit the activity of CAR-T cells and promote tumor escape (27, 31). For example, Kloss et al. (32) designed a type of PSMA-CAR-T cells that simultaneously expressed the dominant-negative TGF-βRII (dnTGF-βRII). In mouse models of human prostatic cancer, these PSMA-CAR-T cells expressing dnTGF-βRII receptors could block TGF-β signaling to promote the proliferation and antitumor activity of CAR-T cells.

#### Remodeling the TME

To improve TME, researchers designed lipid nanoparticles comprising drug cocktail to remodel TME (i.e., blocking the suppressive immune cells in TME and activating the key anti-tumor immune cells, which reverses the adverse TME). After maximizing this effect, tumor-specific CAR-T cells are administered (33). Of note, this therapy significantly improves the curative effect of CAR-T cells in solid tumors.

### Homing Barriers of CAR-T Cells

Homing barriers of CAR-T cells refers to CAR-T cells that cannot efficiently enter solid tumors. CAR-T cells only and effectively kill tumor cells when they migrate to the tumor site, specifically into the interior of the primary tumor and metastasis. There exist 2 major barriers between CAR-T cells and solid tumor cells, including vascular barrier and stromal barrier in solid tumor tissues. Therefore, we can break through these two barriers by taking the measures listed below.

#### Regional Injection of CAR-T Cells

Clinically, CAR-T cells are frequently injected into the body *via* intravenous injection. Nevertheless, due to the barriers between the input CAR-T cells and solid tumor cells, only a small amount of CAR-T cells enter the interior of the solid tumor, most of them remaining in the peripheral circulation. To increase the quantity and concentration of CAR-T cells in the local area, regional injection of CAR-T cells, a safe and effective method can be adopted (34, 35). Katz et al. designed CEA-CAR-T cells for patients diagnosed with unresectable CEA+ adenocarcinoma liver metastases (LM), then injected CEA-CAR-T cells into liver metastases with percutaneous hepatic artery infusions (HAIs). Their results revealed that CAR-T cells could be detected in tumors and normal livers, while not or rarely in peripheral blood. Besides, CEA-CAR-T HAIs demonstrated satisfactory safety (no grade 4 or 5 adverse events) and clinical activity (36).

A few clinical or animal studies have shown that regional injection of CAR-T cells in other solid tumors has a better therapeutic effect. For instance, regional intraperitoneal delivery of TAG72-CAR-T cells for the treatment of ovarian cancer (37); and intracranial infusions of interleukin-13 receptor alpha 2 (IL13Rα2)-CAR-T cells for the treatment of recurrent multifocal glioblastoma (38).

#### Increasing the Penetrability of CAR-T Cells

A large amount of extracellular matrix (ECM) has been observed around solid tumors and between tumor cells. Notably, the primary component of ECM is heparan sulfate proteoglycans (HSPGs). The heparanase (HPSE) degrade HSPGs. Therefore, researchers designed CAR-T cells that expressed HPSE and enhance their ability to degrade HSPGs, improve the penetration of CAR-T cells, and promote homing as well as infiltration of CAR-T cells in solid tumors (39). Similarly, corresponding CAR-T cells can be designed for other matrix components to enhance homing ability in solid tumors.

#### Improving the Ability of CAR-T Cells to Aggregate Around Solid Tumors

Tumor cells secrete chemokines (which act as chemotactic agents for immunosuppressive cells and enhance the immunosuppressive effect of TME), including interleukin-8 (IL-8, CXCL8) (40, 41). Research indicated that CAR-T cells expressing the IL-8 ligand (CXCR2) could migrate more efficiently to sites where IL-8 was present and to tumor cells with IL-8 (42). Interestingly, Smith et al. (43) developed a biopolymer device that could place CAR-T cells and place this device on the surface of a solid tumor, thereby increasing the concentration of CAR-T cells around the solid tumor and more effectively killing tumor cells.

### CAR-T Cell Differentiation, Increased Depletion, Decreased Proliferation, and Short Duration of Activity

CAR-T cell differentiation, increased exhaustion, decreased proliferation, and short duration of activity maintenance, weaken the therapeutic effect of CAR-T cells in tumors. Additionally, the metabolic state of CAR-T cells influences the survival and efficacy of CAR-T cells (44). In contrast with hematologic malignancies, the activity maintenance time of CAR-T cells in patients with solid tumors was significantly shortened. These phenomena have been attributed to several reasons including (a) After injecting CAR-T cells into the body, they recognize, bind to, and kill the target cells, causing depletion of CAR-T cells; (b) Suppressive TME; (c) Selecting specific subsets of T cells to construct CAR-T cells; (d) The design of CAR itself (for example, whether it contains costimulatory molecules and their number); (e) Whether CAR-T cells can be effectively activated in the body; (f) Differentiation type of CAR-T cells. Notably, these reasons can be addressed.

For instance, the infusion of CAR-T cells can be intermittent or in increments to guarantee the concentration and total amount of CAR-T cells in the body. The persistence of CAR-T cells is associated with the curative effect. Selecting specific T cell subtypes to construct CAR-T cells (such as Tn or Tcm) or adopting measures to promote the production of these cells enables CAR-T cells to survive longer with a longer anti-tumor effect in the body (45, 46). A few researchers used different subtypes of T cells to construct CAR-T cells (47). Consequently, they discovered that CAR-T cells constructed with either naive T cells (Tn) or central memory T cells (Tcm) demonstrated greater proliferation, antitumor activity, and longer duration of activity compared to those constructed with effector memory T cells (Tem). Moreover, CAR-T cells in the body gradually differentiated into shorter effector forms *in vivo* (i.e., reduced Tn and Tcm, increased Tem), which was primarily related to the PI3K pathway. Inhibition of PI3K pathway *in vivo* improved the persistence and efficacy of CAR-T cells (48). Further, by optimizing the manufacturing conditions of CAR-T cells (only changing the initial activation conditions and the medium), more Tcm or stem-like memory T cells can be differentiated, thereby prolonging the persistence of CAR-T cells and effectively regulating the growth of tumor cells (49).

### Immune Checkpoints and CRISPR/Cas9 Technology

**Figure 4:**
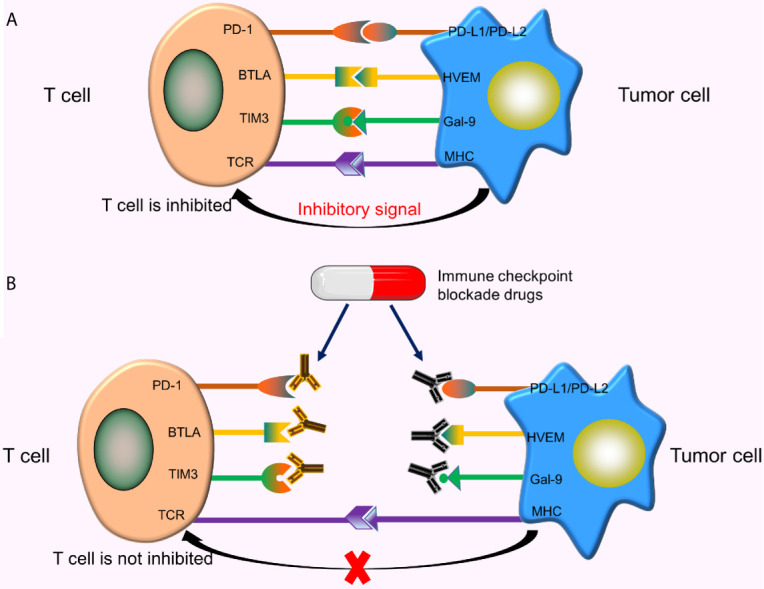
Immune checkpoints and immune checkpoint inhibitors. **(A)** When these immune checkpoints bind to the corresponding ligands, inhibitory signals transmitted from tumor cells inhibit T cell activation; **(B)** These immune checkpoint inhibitors specifically bind to the corresponding checkpoints on the T-cell surface or tumor cell surface, thereby blocking the binding of these checkpoints to corresponding ligands, ultimately preventing the transmission of inhibitory signals from tumor cells to T cells.

The surface of T cells comprises numerous molecules with different types and functions. Some molecules provide activation signals, including costimulatory molecules CD28, 4-1BB. Besides, other molecules provide inhibitory signals, called immune checkpoints. When these immune checkpoints bind to the corresponding ligands, inhibitory signals transmitted from tumor cells inhibit the activation of T cells, resulting in reduced T cell proliferation and tumor-killing effect (**Figure 4A**). As such, tumor cells inhibit T cell activity by activating immune checkpoints. Currently, the immune checkpoints related to CAR-T cells, including programmed death-1 (PD-1) (50), cytotoxic T lymphocyte-associated protein 4 (CTLA-4) (51), T cell immunoglobulin mucin domain 3 (TIM3) (52), B and T lymphocyte attenuator (BTLA) (53), etc. The therapeutic effects of CAR-T cells can be enhanced by using immune checkpoint inhibitors, designing special CARS, or using gene-editing technology.

#### Immune Checkpoint Blockade Drugs and CAR-T Cell Immunotherapy

Common immune checkpoint blockade drugs (immune checkpoint inhibitors) include Nivolumab and Pembrolizumab (PD-1 inhibitors), Atezolizumab and Avelumab (PD-L1 inhibitors), Ipilimumab (a CTLA-4 inhibitor), etc. These inhibitors specifically bind to corresponding checkpoints on the T-cell surface or tumor cell surface, thereby blocking the binding of these checkpoints to the corresponding ligands, and ultimately preventing the transmission of inhibitory signals from tumor cells to T cells (**Figure 4B**). Studies show that the combined use of CAR-T cells and immune checkpoint inhibitors significantly improve the efficacy of CAR-T cells in solid tumors (54).

#### Immune Checkpoint and CAR-T Cell Immunotherapy

Immune checkpoints can be used to design special CARs and improve the function of CAR-T cells. Since PD-L1 and PD-L2 are expressed on the surface of tumor cells, CAR-T cells with chimeric PD-1 can be designed. A few researchers adopted this aiming to reverse design CAR for PD-1/PD-L. PD-1 was expressed on the surface of these CAR-T cells, which recognizes the ligands for the PD-1 receptor expressed in various solid cancers and convert inhibitory signals into activation signals of T cells. Notably, these cells have shown strong antitumor effects in murine models (55). Other researchers designed a type of CAR-T cells. These cells could secrete scFv that blocked PD-1 to protect CAR-T cells from PD-1/PD-L1 inhibition. These cells might exhibit better efficacy and higher safety (56).

#### CRISPR/Cas9 Technology and CAR-T Cell Immunotherapy

Because of low cost, easy operation, and high-efficiency CRISPR/Cas9 technology has evolved as the most widely used gene-editing technology. Eyquem et al. (57) applied CRISPR/Cas9 technology to construct more powerful CAR-T cells, which delayed the differentiation and exhaustion of effector T cells and improved the curative effect of CAR-T cells. Moreover, using CRISPR/Cas9 technology knocked out genes associated with immune checkpoints, preventing surface expression of these immune checkpoints on CAR-T cells, thereby improving the persistence and activity of CAR-T cells (58). For instance, Hu et al. (59) designed CD133-targeted CAR-T cells and knocked out the genes expressing PD-1 with CRISPR/Cas9 technology. Unlike the control group, PD-1-deficient CAR-T cells effectively regulated tumor growth in a mouse model of glioma. The use of CRISPR/Cas9 technology in designing and modifying CAR-T cells will undoubtedly have significant potential in the treatment of solid tumors in the future.

### The Trogocytosis of CAR-T Cells

**Figure 5:**
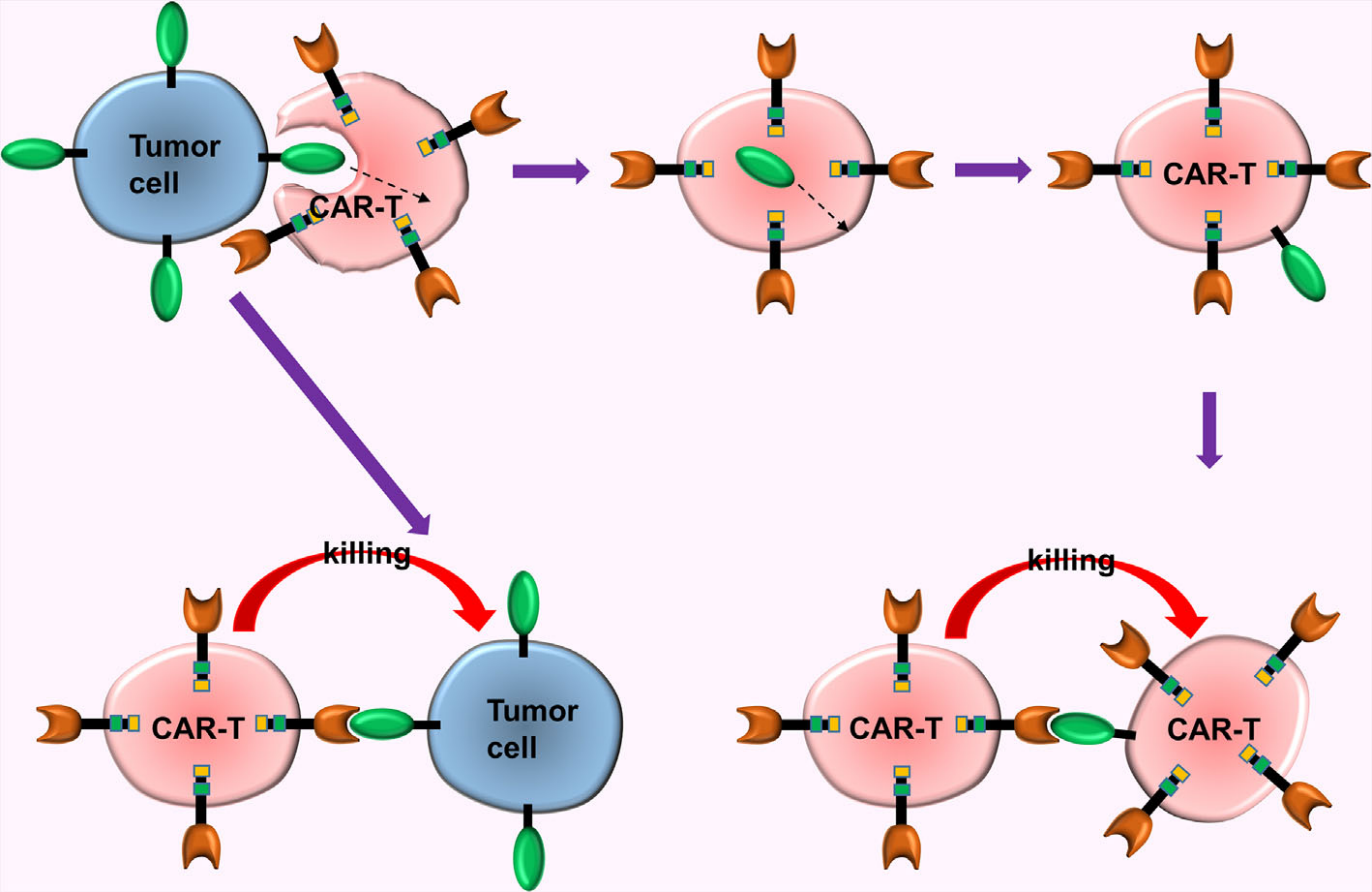
The trogocytosis of CAR-T cells. The CAR-T cells accidentally ingested surface antigens of tumor cells *via* trogocytosis, and transferred these antigens to the surface of CAR-T cells, causing them to attack each other, ultimately causing the depletion of the CAR-T cells.

Mechanisms and reasons for tumor immune escape are complex puzzling scientists for a long time. A new study offers a subversive insight, i.e., the key factor in tumor immune escape in CAR-T turns out to be the CAR-T cells. Hamieh et al. (60) simulated the recurrence process of CD19-CAR-T cells in the treatment of ALL in mouse models with ALL. This study uncovers the mechanism of tumor immune escape in CAR-T cell immunotherapy (60), i.e., (a) The CAR-T cells accidentally ingest the surface antigens of tumor cells *via* trogocytosis, then transfer these antigens to the surface of CAR-T cells, causing them to attack each other, ultimately causing the depletion of the CAR-T cells; (b) When CD19 expression on tumor cell surface is reduced to a certain extent (caused by trogocytosis), the CAR-T cells release inhibitory molecules which prevent cannibalism, and enables tumor cells with low CD19 expression to escape from the chase of the CAR-T cells (**Figure 5**).

Researchers discovered that CAR-CD19-CD28-T cells and CAR-CD19-4-1BB-T cells exhibited different sensitivity to the density of tumor cell surface antigens. Thus, they combined these 2 types of CAR-T cells (with different costimulatory molecules) to counteract the effect of trogocytosis and significantly enhanced the therapeutic effect (60). This mechanism of immune escape might be prevalent in CAR-T, thus trogocytosis of CAR-T cells can be counteracted *via* a combined use of different types of CAR-T cells.

### Tumor Antigen Heterogeneity

Tumor heterogeneity refers to the formation of cell subsets with different biological characteristics in the growth process of tumors after multiple division and proliferation. It is one of the characteristics of malignant tumors and a vital factor in cancer treatment failure, cancer recurrence and drug resistance (61). Also, malignant tumor antigens are highly heterogeneous.

#### Tumor Antigen Heterogeneity Promotes Tumor Immune Escape

For the same patient, even if the tumor at the same site or even the tumor cells on the same tumor may not express the same tumor antigens (62). Besides, the number or types of tumor antigens are different before and after tumor treatment, as well as in the original and recurrent lesions (14, 31, 63). Conventional CAR-T cells only target one tumor surface antigen, thereby allowing tumor cells that do not express or under-express that antigen to escape, ultimately causing tumor recurrence. This phenomenon is similar to that when patients take broad-spectrum antibiotics for a long period, sensitive bacteria are inhibited, while insensitive bacteria or fungus maximize the opportunity to multiply and grow, eventually causing superinfection. Therefore, tumor antigen heterogeneity increases the difficulty of solid tumor treatment and the possibility of tumor recurrence.

#### How to Deal With Tumor Antigen Heterogeneity in CAR-T Cell Immunotherapy

By solving the problem of tumor antigen heterogeneity, the recurrence rate of tumors can be theoretically reduced. The following measures can be adopted to deal with tumor antigen heterogeneity: (a) Identifying and using TSAs as targets for CAR-T cells which is also an ideal method; (b) Using combined CAR-T cell immunotherapy, tandem CARs, or AND gate CARs to cover as much TAAs of tumor cell surface as possible to counteract tumor antigen heterogeneity; (c) Increasing the density of target antigens. For instance, one study revealed that Bryostatin1 (one drug) could up-regulate the expression of CD22 in leukemia cell lines and lymphoma cell lines, thus improving the function and persistence of CAR-T cells in the body (64); (d) Increasing the affinity of CAR-T cells. For example, Drent et al. (65) discovered that the co-stimulator CD28 and 4-1BB were simultaneously used to build a CAR, increasing the affinity of CAR-T cells. This CAR was highly safe significantly improving its anti-tumor performance *in vivo*, and retaining its ability to recognize target antigen density; (e) Optimizing the structure of CAR and reducing the threshold of CAR reaction. Majzner et al. (66) added additional ITAM to CAR to enhance signal strength, hence enabling the CAR-T cells to recognize low-antigen-density tumor cells. Also, the CAR with the addition of a CD28-hinge-transmembrane region reduced the threshold of CAR response, thereby enhancing the activity of CAR-T cells against low-antigen-density tumor cells; (f) Designing the CARs that can recruit other innate immune cells to kill tumor cells e.g., the fourth-generation CARs (67); (g) The universal CARs with characteristics enabling them to target different tumor antigens and effectively fight against tumor antigen heterogeneity.

### Reducing Toxicity by Controlling the Activity of CAR-T Cells

CAR-T cells inevitably produce toxic effects in the treatment of cancer, including CRS, CRES, off-target effects, etc. If the activity of CAR-T cells in the body can be regulated in a timely and appropriate manner to reduce toxicity, the CAR-T will be safer and more widely used. To minimize these toxic reactions, optimizing the production method of CAR-T cells and the structure of CARs is necessary.

#### Transient mRNA-Mediated CAR Expression

Transient mRNA-mediated expression of CAR usually adopts electroporation to transfer the mRNA encoding the target genes into the cytoplasm of T cells (the mRNA does not enter into the nucleus, thus insertion mutations are extremely rare). These mRNAs encode the corresponding proteins eventually expressing corresponding CARs on the surface of T cells. The instability and short survival time of the mRNA can lead to transient expression of the encoded proteins. Therefore, CARs expressed on the surface of T cells are transient, thus improving the safety of CAR-T cells. One study reported that more than 80% of CAR-T cells transfected with electroporation survived, while 94% of these cells were expressed CARs (68).

mRNA electroporation is presently a relatively safe and cost-effective method for T cell gene transduction, and it is more and more widely used in CAR-T cell immunotherapy. Due to the transient nature of the CAR-T cells produced by this method, several rounds of CAR-T cell infusion are required during treatment. Nevertheless, repeated injections of CAR-T cells might induce anaphylaxis (69).

#### Suicide Genes

The addition of suicide genes to CAR-T cells irreversibly induces apoptosis in the CAR-T cells that cause the toxic reaction, ensuring the safety of CAR-T cell immunotherapy (70). There exist 3 commonly used suicide genes, including (a) Herpes simplex virus thymidine kinase (HSV-tk), which phosphorylate ganciclovir (GCV), transforming it into toxic GCV-triphosphate compound, affecting DNA synthesis and ultimately inducing CAR-T cell death (71). Since interfering DNA synthesis to induce T cell death is a gradual process, HSV-TK takes a longer time to eliminate the CAR-T cells (72); (b) The caspase 9 (iCasp9) suicide genes, which can be induced into dimerization by AP1903 (one chemical inducer of dimerization, CID), rapidly inducing CAR-T cells apoptosis and preferentially killing activated CAR-T cells with strong transcriptional activity (73). Notably, this further enhances the safety of CAR-T cell immunotherapy; (c) CD20 and truncated epidermal growth factor receptor (EGFRt), i.e., CAR-T cells co-expressing CD20 or EGFRt are constructed. When severe toxicity occurs during treatment, rituximab (a CD20-targeted drug) and cetuximab (an EGFRt-targeted drug) are injected, respectively, inducing antibody-dependent cell-mediated cytotoxicity (ADCC) effect to eliminate these CAR-T cells (74, 75). The suicide genes are installed in CAR-T cells to eliminate CAR-T cells causing toxic reactions. Despite regulating toxic reactions, they also reduce the number of CAR-T cells and affect the efficacy.

#### On-Switch CAR

**Figure 6:**
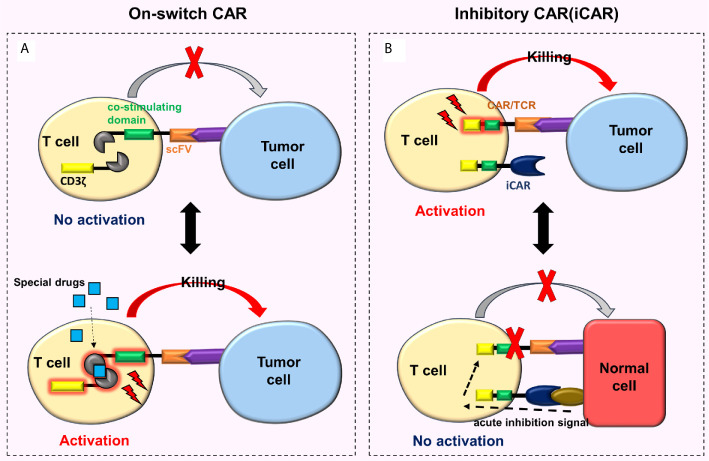
On-switch CAR and iCAR. **(A)** The on-switch CAR-T cells are initially turned off, activated only with specially designed drugs, then recognize, bind to, and kill the targeted tumor cells. When the drugs are not present, the CAR-T cells return to their off state; **(B)** When iCAR-T cells bind to the corresponding tumor cells, iCAR-T cells are activated and kill the tumor cells. Nonetheless, when iCAR-T cells combine with normal cells, the iCARs produce inhibitory signals which prevent traditional CARs or TCRs from functioning, thus the normal cells remain undamaged.

To regulate the activity of CAR-T cells in the body and reduce toxicity, researchers developed an on-switch CAR system (76, 77). This new type of on-switch CAR-T cell is initially in a closed state. Only after the use of specially designed drugs, these cells are activated, then identify, bind and kill the targeted tumor cells (**Figure 6A**). When the drug is not present, CAR-T cells return to their off state, e.g., the on-switch CAR designed by Wu et al. (76), whose the intracellular signal domain was divided into two separate units (i.e., the co-stimulating domain and CD3ζ). The co-stimulating domain and CD3ζ can only be reassembled in the presence of a heterodimerizing small molecule (rapamycin analog AP21967). At this time, the CAR-T cells are activated. By adjusting the dosage of AP21967, time, location, and dose of CAR-T cell activity *in vivo* can be precisely regulated, thereby reducing the toxicity.

#### Inhibitory CAR (iCAR)-T Cell

Fedorov et al. (78) designed a special CAR-T cell called iCAR-T cell that reduces off-target effects. This cell surface simultaneously expresses traditional CARs and iCARs or traditional TCRs and iCARs (**Figure 6B**). The conventional CARs or TCRs target tumor cells, and the iCARs target normal cells. The iCAR comprises a surface antigen recognition area (which recognizes the normal cells) and an acute inhibition signal area (based on CTLA-4 or PD-1). When the iCAR-T cell binds to the corresponding tumor cell, it will be activated and then kills the tumor cell. However, when the iCAR-T cell combines with the normal cell, iCAR generates inhibitory signals preventing traditional CARs or TCRs on the surface of an iCAR-T cell from functioning, hence the normal cell remains undamaged. Moreover, this effect is reversible. When these cells meet and bind to the targeted tumor cells again, they can still be activated and kill the tumor cells. These characteristics enable iCAR-T cells to distinguish tumor cells from normal cells (78). Although research on iCAR is extremely rare, its design theory is feasible and innovative. One of the major difficulties is the need to identify tissue-specific antigens for iCAR that are missing or down-regulated in tumors but highly expressed in normal tissues.

### Other Improved Methods of CAR-T Cell Immunotherapy

In recent years, CAR-T has rapidly developed. In addition to the above-mentioned coping strategies in the treatment of solid tumors, many other improved methods have been reported, for example, CAR-T cells combined with tumor vaccine treatment (79); CAR-T cells combined with radiotherapy or chemotherapy (80, 81); CAR-T cells combined with oncolytic viruses (82); CAR-T cells combined with photothermal therapy (83); CAR-T cells combined with nanotechnology (84), etc.

## Economic Restriction

CAR-T cell immunotherapy is extremely expensive. The currently available 2 CAR-T cell immunotherapy in the market, i.e., Kymriah (Novartis) and Yescarta (Kite Pharma), have an average expected cost of $510,963 and $402,647 per patient, respectively (85) (conservative estimate). The high cost significantly limits the development and clinical application of CAR-T cell immunotherapy. Therefore, reducing its research and development expenses and clinical use costs is a major problem at the moment

At present, the manufacturing process of CAR-T cells is still in the “manual stage”, where most operations need to be manually completed, therefore, the output is limited by expensive cost. At the 3^rd^ Global CAR-TCR Summit in 2017, experts believed that accelerating automated production is vital to reducing the cost of CAR-T cell immunotherapy. As such, there is an urgent need to upgrade the CAR-T cell production process. Notably, automatic, fully enclosed, and cGMP (current good manufacture practices)-compliant CAR-T cell preparation is a major trend in the future (86, 87). Meanwhile, other approaches to reducing the cost of CAR-T cells have been reported. For example, electroporation technology significantly shortens the production time of CAR-T cells, reducing the production cost, with a high curative effect and few adverse reactions (88). Shortening the time of *in vitro* culture (can be shortened to 3 days) prevents the differentiation of CAR-T cells during amplification (i.e., the reduction of Tscm memory stem cells is prevented), which is simple, cost-effective, and most importantly, it significantly improves the proliferation ability and tumor-killing effect of CAR-T cells (89). Additionally, TSAs on the surface of tumor cells are the most ideal targets for CAR-T cells, however, identifying new TSAs remains difficult. Next-generation sequencing technologies are usually used to screen neoantigens (90), nevertheless, they have a high screening cost. As such, known TAAs can be used for in-depth research.

## Prospect and Summary of CAR-T Cell Immunotherapy

As an emerging tumor immunotherapy, CAR-T cell immunotherapy has a remarkable development and significant potential in the treatment of tumors. Besides, it has demonstrated notable future opportunities in the mainstream cancer treatment of solid tumors. CAR-T cell immunotherapy still has numerous obstacles in the treatment of solid tumors, including the lack of specific targets, TME inhibition, CAR-T cells homing obstacle, the trogocytosis of CAR-T cells, etc. Nonetheless, with the continuous breakthrough of these problems, CAR-T cell immunotherapy will exert better curative effects in the treatment of solid tumors, and might even be one of the primary treatment options in the future.

## Author Contributions

LM: Writing-Original draft preparation, Investigation, and figure preparation. ZZ: Investigation and figure preparation. ZR: Investigation. FT: Investigation. YL: Conceptualization, Methodology, Supervision. All authors contributed to the article and approved the submitted version.

## Funding

This work was supported by Special Research Project of Lanzhou University Serving the Economic and Social Development of Gansu Province (054000282), Lanzhou Talent Innovation and Entrepreneurship Project (2020-RC-38), and Fundamental Research Funds for the Central Universities (lzujbky-2020-kb14).

## Conflict of Interest

The authors declare that the research was conducted in the absence of any commercial or financial relationships that could be construed as a potential conflict of interest.
